# Global dataset of soil eukaryotic communities created with a uniform protocol and long read sequencing

**DOI:** 10.1038/s41597-026-07315-y

**Published:** 2026-05-05

**Authors:** Vladimir Mikryukov, Olesya Dulya, Kessy Abarenkov, Sten Anslan, Niloufar Hagh-Doust, Victoria Prins, Kristel Panksep, Sergei Põlme, Khalid S. Ibrahim, Mo Bahram, Kalev Adamson, Ahto Agan, Talaat Ahmed, Juha M. Alatalo, Felipe E. Albornoz, Abdullah MS Al-Hatmi, Saad Alkahtani, Julieta Alvarez-Manjarrez, Jelena Ankuda, Alexandre Antonelli, Manikandan Ariyan, Kęstutis Armolaitis, Farzad Aslani, Isabel C. Barrio, Marijn Bauters, Elisabeth Machteld Biersma, Krišs Bitenieks, Gregory Bonito, Francis Q. Brearley, Kari Anne Bråthen, Franz Buegger, Klaus Butterbach-Bahl, Miklós Bálint, Erin K. Cameron, Fabiana Canini, Rebeca Casique-Valdés, Adriana Corrales, Evgeny A. Davydov, Eske De Crop, André De Kesel, Joseph Fovo Djeugap, Rein Drenkhan, Camila Duarte Ritter, Sergey V. Dudov, Mikk Espenberg, Ongua Fanuel, Vladimir E. Fedosov, Luke Florence, Brendan R. Furneaux, Ariadne N. M. Furtado, Sanni Färkkilä, Natalia S. Gamova, Roberto Garibay-Orijel, József Geml, Soumya Ghosh, Roberto Godoy, Daniyal Gohar, Marieka Gryzenhout, Ayad H. Hasan, Amr H. Hashem, Jacob Heilmann-Clausen, Terry W. Henkel, Indrek Hiiesalu, Inga Hiiesalu, Mahdieh S. Hosseyni Moghaddam, Kevin D. Hyde, Karina Inostroza, Khalil Kariman, Elina Karimullina, Sebastian Kepfer-Rojas, Abdul Nasir Khalid, Darta Klavina, Petr Kohout, Yuri N. Korotkov, John Y. Kupagme, Olavi Kurina, Louis James Lamit, Adebola Azeez Lateef, Njouonkou André Ledoux, Young Woon Lim, Jose G. Maciá-Vicente, Kristaps Makovskis, Sebastián Martínez, César Marín, Peter Meidl, Peter E. Mortimer, Sunil Mundra, Victoria Naluyange, Tarquin Netherway, Kevin K. Newsham, Eduardo Nouhra, Casper Nyamukondiwa, Vincent Nteziryayo, Dennis M. W. Ochieno, Jane Oja, Vladimir G. Onipchenko, Eveli Otsing, Mustafa Nadhim Owaid, Meike Piepenbring, Polina Pochekutova, Maihyra Marina Pombo, Karin Pritsch, Rasmus Puusepp, Jaan Pärn, Kadri Põldmaa, Saleh Rahimlou, Andrea C. Rinaldi, Oscar Rojas, Tomas Roslin, Kadri Runnel, Elisabeth Rähn, Malka Saba, Alessandro Saitta, Talal Sabhan Salih, Joosep Sarapuu, Eduard Serrano, Oscar Serrano, Dipon Sharmah, Cathy Sharp, Maria W. Skalska-Tuomi, Kassim Issifou Tchan, Camille Truong, Helga van der Merwe, Linda L. P. Vanié-Léabo, Aida M. Vasco-Palacios, Annemieke Verbeken, Lukáš Vlk, Nalin N. Wijayawardene, Jennifer L. Wood, W. A. Erandi Yasanthika, Nourou S. Yorou, Geoffrey Zahn, Irma Zettur, Laura Zucconi, Urmas Kõljalg, Leho Tedersoo

**Affiliations:** 1 Department of Botany, Institute of Ecology and Earth Sciences, University of Tartu, Juhan Liivi 2, Tartu, 50409 Estonia; 2 Mycology and Microbiology Center, University of Tartu, Juhan Liivi 2, Tartu, 50409 Estonia; 3 Natural History Museum, University of Tartu, Vanemuise 46, Tartu, 51003 Estonia; 4 Department of Biological and Environmental Science, Faculty of Mathematics and Science, University of Jyväskylä, Survontie 9 C, Jyväskylä, Central Finland 40500 Finland; 5 Institute of Technology, Faculty of Science and Technology, University of Tartu, Nooruse 1, Tartu, 50411 Estonia; 6 Department of hydrobiology and fisheries, Institute of Agricultural and Environmental Sciences, Estonian University of Life Sciences, F. R. Kreutzwaldi 1, Tartu, 51006 Estonia; 7 Institute of Freshwater Research, Department of Aquatic Resources, Swedish University of Agricultural Sciences, Stångholmsvägen 2, Drottningholm, 178 93 Sweden; 8 Natural History Museum and Botanical Garden, University of Tartu, Vanemuise 46, Tartu, 50410 Estonia; 9 Department of Biology, College of Science, University of Zakho, Zakho, Kurdistan Region 42002 Iraq; 10 Department of Agroecology, Aarhus University, Forsøgsvej 1, Slagelse, 4200 Denmark; 11 Department of Ecology, Swedish University of Agricultural Sciences (SLU), Ulls väg 18B, Uppsala, 75651 Sweden; 12 Chair of Silviculture and Forest Ecology, Institute of Forestry and Engineering, Estonian University of Life Sciences, F. R. Kreutzwaldi 5, Tartu, 51006 Estonia; 13 Environmental Science Center, Qatar University, Doha, Qatar; 14 School of Biological Sciences, The University of Western Australia, 35 Stirling Hwy, Crawley, WA 6009 Australia; 15 Natural and Medical Sciences Research Center, University of Nizwa, Nizwa, 616 Oman; 16 Department of Zoology, College of Science, King Saud University, P.O. Box 2455, Riyadh, 11451 Saudi Arabia; 17 Micología Integral, Instituto de Biología, Universidad Nacional Autónoma de México, Tercer circuito interior s/n, Ciudad Universitaria, Coyoacán, Mexico City, 04510 México; 18 Vokė Branch, Institute of Agriculture, Lithuanian Research Centre for Agriculture and Forestry, Žalioji Sq. 2, Vilnius, LT-02232 Lithuania; 19 Royal Botanic Gardens, Kew GB, Richmond, Surrey, TW9 3AE UK; 20 Gothenburg Global Biodiversity Centre, Department of Biological and Environmental Sciences, University of Gothenburg, Göteborg, 405 30 Sweden; 21 Department of Biology, University of Oxford, South Parks Road, Oxford, OX1 3RB UK; 22 Department of Silviculture and Ecology, Institute of Forestry, Lithuanian Research Centre for Agriculture and Forestry, Liepų str. 1, Girionys, Kaunas distr. LT-53101 Lithuania; 23 Above-belowground interactions group, Institute of Biology, Leiden University, Rapenburg 70, Leiden, Netherlands; 24 Faculty of Life and Land, Agricultural University of Iceland, Árleyni 22, Reykjavík, Iceland 112 Iceland; 25 Q-ForestLab, Department of Environment, Ghent University, Coupure Links 653, Gent, 9000 Belgium; 26 Natural History Museum of Denmark, Øster Voldgade 5-7, Copenhagen K, 1350 Denmark; 27 Latvian State Forest Research Insititute “Silava”, Rīga str. 111, Salaspils, LV-2169 Latvia; 28 Department of Plant Soil and Microbial Sciences, Michigan State University, Michigan, MI 48824 USA; 29 Department of Natural Sciences, Manchester Metropolitan University, Chester Street, Manchester, M1 5GD UK; 30 Departiment of Arctic and Marine Ecology, UiT The Arctic University of Norway, Breivika, Tromsø, 9037 Norway; 31 Research Unit Environmental Simulation, Helmholtz Zentrum München, Ingolstädter Landstraße 1, Neuherberg, 85764 Germany; 32 Agroecology, Department, Aarhus University, Ole Worms Alle 3, Aarhus, 8000 Denmark; 33 Functional Environmental Genomics, Senckenberg Biodiversity and Climate Research Centre, Georg-Voigt-Str. 14-16, Frankfurt am Main, Hesse 60325 Germany; 34 Institute of Insect Biotechnology, Justus Liebig University, Heinrich-Buff-Ring 26, Gießen, Hesse 35392 Germany; 35 Functional Environmental Genomics, LOEWE Centre for Translational Biodiversity Genomics, Senckenberganlage 25, Frankfurt am Main, Hesse 60325 Germany; 36 Department of Biology, Saint Mary’s University, 923 Robie St, Halifax, Nova Scotia B3H 3C3 Canada; 37 Department of Ecological and Biological Sciences, University of Tuscia, Largo dell’Università, Viterbo, 01100 Italy; 38 Horticulture department, Universidad Autónoma Agraria Antonio Narro, Saltillo, Coahuila 25315 México; 39 Society for the Protection of Underground Networks (SPUN), 500 South DuPont Highway, Dover, DE 19901 USA; 40 no affiliation, Barnaul, Russia; 41 Department of Biology, Ghent University, K.L. Ledeganckstraat 35, Gent, 9000 Belgium; 42 Research Department, Biodiversity and Evolution, Meise Botanic Garden, Nieuwelaan 38, Meise, B-1860 Belgium; 43 Department of Crop Sciences, Plant Protection, University of Dschang, Dschang, West Region PO. Box 222, Cameroon; 44 Intituto Juruá, Manaus, Amazonas 69083-300 Brazil; 45 no affiliation, Moscow, Russia; 46 Department of Geography, Institute of Ecology and Earth Sciences, University of Tartu, Vanemuise 46, Tartu, 51003 Estonia; 47 Soils, Environment and Agro-meteorology, National Agricultural Research Laboratories-Kawanda, National Agricultural Research Organization NARO, Kampala, 7065 Uganda; 48 Department of Ecological, Plant and Animal Sciences, La Trobe University, Bundoora, Victoria 3086 Australia; 49 Department of Biomedical Sciences, University of Cagliari, Cittadella Universitaria, Monserrato, CA I09042 Italy; 50 University of Tartu, Ülikooli 18a, Tartu, 51005 Estonia; 51 Universidad Nacional Autonoma de Mexico, Instituto de Biologia, Circuito exterior s/n, Ciudad de Mexico, Ciudad de, Mexico, 4510 México; 52 Environmental Microbiome Research Group, Research and Development Centre, Eszterházy Károly Catholic University, Leányka u. 8, Eger, 3300 Hungary; 53 University of the Free State, Bloemfontein, 9301 South Africa; 54 Instituto de Ciencias Ambientales y Evolutivas, Universidad Austral de Chile, Isla Teja sn, Valdivia, 5090000 Chile; 55 Department of Botany and Plant Pathology, Oregon State University, 2701 SW Campus Way, Corvallis, OR 97331 USA; 56 Department of Genetics, Natural and Agricultural Sciences, University of the Free State, Bloemfontein, 9300 South Africa; 57 Department of Medical Microbiology, Faculty of Science and Health, Koya University, Koya, 44023 Kurdistan Region – F.R. Iraq; 58 Botany and Microbiology Department, Faculty of Science, Al-Azhar University, Nasr City, Cairo, 11884 Egypt; 59 Center for Macroecology, Evolution and Climate, Globe institute, University of Copenhagen, Universitetsparken 15, Copenhagen, 2100 Denmark; 60 Department of Biological Sciences, California State Polytechnic University, Humboldt, Arcata, CA 95521 USA; 61 Institute of Ecology and Earth Sciences, University of Tartu, Juhan Liivi 2, Tartu, 50409 Estonia; 62 Department of Botany and Microbiology, College of Science, King Saud University, P.O. Box 2455, Riyadh, 11495 Saudi Arabia; 63 Center of Excellence in Fungal Research, Mae Fah Luang University, Chiang Rai, 57100 Thailand; 64 BIOSFERA Research & Conservation, DS Can Mutxo, Sils, Girona, 17410 Spain; 65 UWA School of Agriculture and Environment, The University of Western Australia, Perth, WA 6009 Australia; 66 Microbiology, Immunology and Infectious Diseases, University of Calgary, 3330 Hospital Drive NW, Calgary, Alberta T2N 4N1 Canada; 67 Geosciences and Natural Resource Management, University of Copenhagen, Rolighedsvej 23, Frederiksberg, 1958 Denmark; 68 Institute of Botany, University of the Punjab, Lahore, Punjab 54320 Pakistan; 69 Laboratory of Microbial Ecology and Biogeography, Institute of Microbiology, Czech Academy of Science, Videnska 1083, Prague, 14220 Czechia; 70 no affiliation, Tankhoi, Russia; 71 Chair of Biological Diversity and Nature Tourism, Institute of Agricultural and Environmental Sciences, Estonian University of Life Sciences, F. R. Kreutzwaldi 5, Tartu, 51006 Estonia; 72 Department of Biology, Syracuse University, 107 College Place, Syracuse, NY 13244 USA; 73 Department of Plant Biology, Faculty of Life Sciences, University of Ilorin, Ilorin, Kwara 1515 Nigeria; 74 Department of Forest Sciences, Faculty of Forestry and Agriculture, University of Helsinki, Agnes Sjöbergin katu 2, P.O. Box 66, Helsinki, 14 Finland; 75 Department of Plant Sciences, Faculty of Science, The University of Bamenda, Bambili, North-West Region PO Box 39, Cameroon; 76 School of Biological Sciences, College of Natural Science, Institue of Biodiversity, Gwanak-ro 1, Seoul, 8826 South Korea; 77 Department of Marine Sciences and Applied Biology, University of Alicante, Alicante, 03080 Spain; 78 Laboratorio de Patología Vegetal, Instituto Nacional de Investigación Agropecuaria, Ruta 8, Km 281, Treinta y Tres, 33000 Uruguay; 79 Centro de Investigación e Innovación para el Cambio Climático (CiiCC), Universidad Santo Tomás, Av. Ramón Picarte 1130, Valdivia, 5090000 Chile; 80 Amsterdam Institute for Life and Environment, Section Ecology & Evolution, Vrije Universiteit Amsterdam, de Boelelaan 1085, Amsterdam, 1081 HV Netherlands; 81 Soil Ecology, Free University Berlin, Berlin, 13357 Germany; 82 Soil Science, Stellenbosch University, PO BOX X1, Matieland, Stellenbosch, Western Cape 7600 South Africa; 83 Applied Symbiotics, 111 9th Road, Hyde Park, Gauteng, 2196 South Africa; 84 Department of Biology, College of Sceince, United Arab Emirates University, Al Ain, Abu Dhabi, UAE; 85 Khalifa Center for Genetic Engineering and Biotechnology, United Arab Emirates University, Al Ain, Abu Dhabi, UAE; 86 Department of Land Use Management, School of Agriculture, Veterinary Sciences and Technology, Masinde Muliro University of Science and Technology, Kakamega, 190-50100 Kenya; 87 British Antarctic Survey, Natural Environment Research Council, Madingley Road, Cambridge, CB3 0ET UK; 88 CONICET-FCEFyN, National University of Córdoba, Av. Vélez Sarsfield 1666, Córdoba, Córdoba, X5016GCN Argentina; 89 Department of Biological Sciences and Biotechnology, Botswana International University of Science and Technology, P. Bag 16, Palapye, Botswana; 90 Centre for Environmental Policy, Imperial College London, Kennedy Building, Silwood Park, Ascot, Berkshire, SL5 7PY UK; 91 Food Science and Technology, Faculty of Agriculture and Bio-engineering, University of Burundi, Avenue l’Unesco no 2, Bujumbura, Burundi 2940 Burundi; 92 Department of Biological Sciences, School of Natural Sciences, Masinde Muliro University of Science and Technology, Kakamega-Webuye Road, Kakamega, 254 Kenya; 93 Biological Faculty, Shenzhen MSU-BIT University, Shenzhen, 518115 China; 94 Department of Environment, College of Applied Sciences-Hit, University Of Anbar, Hit, Anbar, Iraq; 95 Mycology, Goethe University, Max-von-Laue-Str. 13, Frankfurt am Main, Hesse, 60438 Germany; 96 Departamento de Botânica, Divisão de Biodiversidade, Instituto Nacional de Pesquisa da Amazônia, Manaus, Amazonas 69067-375 Brazil; 97 Department of Plant Pathology and Environmental Microbiology, Pennsylvania State University, University Park, State College, PA 16802 USA; 98 Freshwater Biology Section, Department of Biology, University of Copenhagen, Universitetsparken 4, Copenhagen, 2100 Denmark; 99 Ecosystems and Environment Research Programme, Faculty of Biological and Environmental Sciences, University of Helsinki, Viikinkaari 1, Helsinki, FI-00014 Finland; 100 Department of Zoology, Institute of Ecology and Earth Sciences, University of Tartu, Juhan Liivi 2, Tartu, 50409 Estonia; 101 Department of Plant Sciences, Quaid-i-Azam University, Islamabad, 45320 Pakistan; 102 Department of Agricultural, Food and Forest Sciences, University of Palermo, Viale Delle Scienze, Palermo, 90123 Italy; 103 Department of Medical Physics, College of Science, University of Mosul, Mosul, Iraq; 104 Estonian Museum of Natural History, Lai 29a, Tallinn, 10133 Estonia; 105 Marine ecology, Centre for Advanced Studies of Blanes, CSIC, Carrer Accés Cala Sant Francesc, 14, Blanes, Girona, 17300 Spain; 106 Department of Botany, Jawaharlal Nehru Rajkeeya Mahavidyalaya, Pondicherry University, Port Blair, Andaman and Nicobar Islands 744101 India; 107 Natural History Museum of Zimbabwe, cnr Park Road & Leopold Takawira Avenue Centenary Park, Bulawayo, Zimbabwe; 108 Department of Geographical and Historical Studies, University of Eastern Finland, Yliopistokatu 7, Joensuu, 80101 Finland; 109 Department of Arctic and Marine Biology, UiT The Arctic University of Norway, Framstredet 39, Tromsø, 9019 Norway; 110 Department of Forestry and Wildlife Management, Science agronomique Kigani-Dada de Kpéssou, Private Agricultural University, Parakou, Benin; 111 Royal Botanic Gardens Victoria, Melbourne, VIC 3004 Australia; 112 School of BioSciences, University of Melbourne, Parkville, VIC 3010 Australia; 113 South African Environmental Observation Network, Arid Lands Node, 97 Memorial Road, Kimberley, 8036 South Africa; 114 Department of Biological Sciences, University of Cape Town, Cape Town, 7701 South Africa; 115 UFR Biosciences, Université Félix Houphouet-Boigny, Abidjan, BPV34 Côte d’Ivoire; 116 Grupo BioMicro, Escuela de Microbiología, Universidad de Antioquia UdeA, Calle 70 No. 52-2, Medellin, 50010 Colombia; 117 Department of Invasion Ecology, Institute of Botany of the Czech Academy of Sciences, Zámek 1, Průhonice, 25243 Czechia; 118 Center for Yunnan Plateau Biological Resources, Protection and Utilization & Yunnan International Joint Laboratory of Fungal Sustainable Utilization in South and Southeast Asia, Biology and Food Engineering, Qujing Normal University, Qujing, Yunnan 655011 China; 119 High-Value Food from Mushrooms and Bioactive Plants in the Green Economy Value Chain Research Group, The Institute of Biotechnology and Genetic Engineering, Chulalongkorn University, 254 Phayathai Road, Pathumwan 10330 Bangkok, Thailand; 120 Department of Microbiology, Anatomy, Physiology and Pharmacology, La Trobe University, Bundoora, VIC 3086 Australia; 121 Tropical Mycology and Plant-Soil fungi Interactions (MyTIPS), Faculty of Agronomy, University of Parakou, Parakou, Benin; 122 Applied Science Department, William & Mary, 540 Landrum Drive, Williamsburg, VA 23185 USA; 123 Mycology and Microbiology Center, Institute of Technology, University of Tartu, Nooruse 1, Tartu, 50411 Estonia

## Abstract

Soil eukaryotes, including fungi, protists, plants, and animals, are central to biosphere functioning and resilience. The Global Standardised Soil Eukaryome Dataset (GloSED) is the first dataset encompassing the entire spectrum of soil eukaryotes, covering 4,063 sampling sites in 121 countries on all continents, revealing nearly one million operational taxonomic units. All samples were collected and analysed using a standardised protocol minimizing technical biases. Long-read sequencing of full-length ITS and 18S-V9 regions provide broad taxonomic coverage and high-resolution identification supported by specialist curation of “dark taxa”. A rigorous bioinformatic processing ensures against homopolymer errors, PCR-mediated chimeras, and index switching providing high data quality. The dataset is supported by raw sequences and an open-source containerised workflow for reproducible analyses. The samples are accompanied by land-cover description and directly measured soil pH, δ^13^C, δ^15^N, as well as P, K, Ca, Mg, and total C and N contents. GloSED is the first database that enables ecological and biogeographic studies of entire soil eukaryotic communities from local to global scales.

## Background & Summary

Soils harbor an extraordinary diversity of eukaryotic life that underpins the functioning of terrestrial ecosystems. Soil-dwelling eukaryotes, including plants, fungi, animals, and diverse groups of protists, are important drivers of nutrient cycling, carbon storage, and ecosystem functioning, stability, and resilience^1^. Over the past decade, global datasets based on environmental DNA sequencing that mapped the diversity and distribution of soil eukaryotes have focused only on individual functional groups or taxa at kingdom, phylum or class levels: fungi in general^2–5^ and mycorrhizal fungi in particular^6^, dominant protist taxa^7,8^, nematodes^9^, earthworms^10^, and springtails^11^, among others.

Despite remarkable progress in mapping specific groups, no global dataset has yet captured the full spectrum of eukaryotes in soil samples. Soil organisms, however, do not operate in isolation; instead, they form complex interaction networks, including predation, parasitism, competition, and symbiosis^1,12^. The need to study these groups together within an integrated framework has long been recognised^12–14^. Yet, cross-group comparisons demand standardized protocols, the use of universal metabarcoding primers and curation of taxonomic annotations by specialists in many taxa. Due to these challenges, a globally standardized dataset spanning multiple eukaryotic kingdoms has not yet been produced, limiting the ability to conduct integrated analyses across taxonomic and functional groups.

Some existing databases, however, offer the potential for expansion. The Global Soil Mycobiome Consortium (GSMc), established in 2014, initially produced a comprehensive dataset of global soil fungal diversity^5^. GSMc employs long-read sequencing of universal eukaryotic primers – full-length internal transcribed spacer (ITS) and partial 18S rRNA gene (SSU) regions – enabling a taxonomic expansion beyond fungi. Here, we leveraged this capability to encompass a broader range of soil eukaryotes, including fungi, protists, animals, and plants. To achieve this, we (i) reprocessed all sequences using an improved bioinformatic workflow to enhance data accuracy; (ii) increased taxonomic resolution by applying the curated EUKARYOME reference database^15^; and (iii) incorporated 1059 new sampling sites, extending the dataset’s geographical coverage from 108 to 121 countries.

Here we present a globally representative dataset of soil eukaryotic diversity – GloSED (Global Standardised Soil Eukaryome Dataset). GloSED inherits from its predecessor, the GSMc database, the key advantage of methodological consistency across geographic regions, achieved through standardised sampling and analytical protocols. The dataset is accompanied by soil chemical and habitat metadata and is supported by raw long-read sequence data and a fully automated open-source bioinformatics pipeline that runs in a standardised, portable software environment, ensuring transparency, reproducibility, and flexible user customization.

## Methods

### Sampling and sample pre-processing

Soil samples were collected following a standardised approach^2^. Each sampling plot comprised a 50 × 50 m square or a 56 m diameter circular area (2500 m²). Within each plot, 40 soil cores (5 cm diameter, 5 cm depth) were collected, with cores taken in pairs on opposite sides of randomly selected trees 1.5 m from the tree trunk (in forests) or at random locations (in non-forested ecosystems). Sampling points were positioned at least 8 m apart to ensure spatial independence while providing comprehensive plot coverage.

Individual soil cores were pooled by combining equal volumes (approximately 25% of the total volume per core), thoroughly mixed, and air-dried within 24 hours of collection. Dried samples were manually homogenised through vigorous rubbing in sealed plastic bags. After homogenization, approximately 30–50 g of the finest material was retained for further analyses. Samples were either shipped to the University of Tartu, Estonia, with silica gel for centralised processing or processed immediately in contributor laboratories following standardised protocols described below.

All sampling was conducted under appropriate national permits and followed local regulations for soil collection. The DNA extracts and soil samples are stored in the Collections of DNA and environmental samples (TUE) of the Natural History Museum at the University of Tartu.

### Soil chemical analyses

Soil pH was measured potentiometrically in 1 M KCl extract at a 1:2.5 soil:solution ratio. Available phosphorus and potassium were measured in 1 M ammonium lactate extracts by flow injection analysis using a Tecator autoanalyser (method ASTN 9/84). Exchangeable magnesium and calcium were determined in 1 M ammonium acetate extracts by flow injection analysis (method ASTN 90/92). At least 0.1 g of ball-milled soil was analysed for total carbon, ^13^C, total nitrogen and ^15^N content using an elemental analyzer coupled with an isotope ratio mass spectrometer in 2–6 replicates. δ^13^C and δ^15^N were obtained following international standards^16^.

### Molecular analyses

DNA extraction was performed from 2 g of homogenised dry soil using the PowerMax Soil DNA Isolation kit (Qiagen, Carlsbad, CA, United States) following the manufacturer’s protocols. Extracted DNA was further purified using the FavorPrepTM Genomic DNA Clean-Up kit (Favorgen, Vienna, Austria) to remove inhibiting compounds and improve amplification success.

Polymerase chain reaction (PCR) was used to amplify the full internal transcribed spacer (ITS) region and 18S-V9 variable region using universal eukaryotic primers ITS9mun (5′-GTACACACCGCCCGTCG-3′) and ITS4ngsUni (5′-CGCCTSCSCTTANTDATATGC-3′)^17,18^. This primer combination amplifies nearly all known eukaryotes with minimal taxonomic bias^19^, excluding only Microsporidia due to primer mismatches. Each primer pair was labeled with identical 12-base indices selected from 115 combinations to minimize index-switching artefacts (Hamming distance between each pair of indices was no less than 3).

PCR reactions contained 5 µl of 5× HOT FIREPol Blend Master Mix (Solis Biodyne, Tartu, Estonia), 0.5 µl each of forward and reverse primers (20 µM), 1 µl DNA extract, and 18 µl ddH₂O in 25 µl total volume. Thermal cycling comprised initial denaturation at 95 °C for 15 min, followed by 25–30 cycles of denaturation (30 s at 95 °C), annealing (30 s at 57 °C), and elongation (1 min at 72 °C), with final extension at 72 °C for 10 min. PCR products were checked on 1% agarose gels for 600–800 bp amplicons. Samples failing initial amplification were re-amplified using 28–38 cycles.

Library preparation and sequencing were performed at the Norwegian Sequencing Centre, University of Oslo, Norway. PacBio SMRTbell libraries were prepared following the manufacturer’s protocols and sequenced on Sequel II instruments using Sequel II Binding kit 2.1, sequencing chemistry 2.0, with 15-hour movie times and 20-minute pre-extension periods. Samples producing <2000 reads after the first sequencing attempt were re-amplified and re-sequenced (this applied to 929 samples). For these samples, reads from repeated runs were combined in the final dataset (in raw sequencing data, reads are provided in independent files for each sequencing attempt), and 97.8% reached the minimum target depth. Sequencing was distributed across 122 SMRT cells.

### Bioinformatics pipeline

GloSED was analysed using the fully automated bioinformatics pipeline NextITS v.1.0.0^20^ (DOI:10.5281/zenodo.15074881) implemented with the workflow manager Nextflow v.25.04.6^21^ and run in standardised, portable software environments (Docker and Singularity containers)^22,23^ to ensure reproducibility. The open-source NextITS pipeline is freely available as a command-line workflow at https://Next-ITS.github.io/ and is also distributed through the cross-platform PipeCraft2 application^24^ (https://pipecraft2-manual.readthedocs.io/en/latest/), which provides a graphical user interface for running the pipeline.

Raw reads were processed through circular consensus sequence (CCS) generation using SMRT Tools (Pacific Biosciences) with minimum pass requirements of 3 and an accuracy threshold of 0.99. Demultiplexing was performed using LIMA v.2.12.0 (Pacific Biosciences) with ‘--min-score 93’ settings for precise index identification. Quality filtering removed sequences with >4 ambiguous nucleotides, >0.01% expected errors^25^, and homopolymer repeats longer than 25 nucleotides. In addition, reads lacking both primer sites in the correct orientation were discarded after primer trimming with cutadapt v.5.0^26^. Full-length ITS regions were then retrieved by ITSx v.1.1.3^27^ targeting all eukaryotes using the updated hidden Markov model (HMM) profile database (provided by R. Henrik Nilsson, University of Gothenburg, Sweden). For sequence processing, we used SeqKit2 v.2.9.0^28^. When selecting representative ITS sequences after ITSx‐based extraction, preference was given to the sequence with the highest average Phred score. Chimeras were detected in a two-step scheme with VSEARCH v.2.29.4^29^: an initial *de novo* detection using the UCHIME algorithm^29^ and a maximum chimera score of 0.6^30^ followed by reference-based verification against the EUKARYOME v.1.9.4^15^ database, with any sequence flagged in either step being excluded. Within each sample, homopolymer correction was performed using the algorithm implemented in NextITS. Within each sequencing run, index-switch (tag-jump) artefacts were removed using the UNCROSS2 algorithm^31^ with the parameter f = 0.01. Prior to clustering, ITS sequences shorter than 250 bp or containing more than 0.6 expected errors per 100 bp were discarded, and the remaining reads were denoised using the UNOISE3 algorithm^32^ with parameters alpha = 6 and minsize = 1, which retains singleton sequences. Surviving denoised reads were then clustered at 98% pairwise similarity using VSEARCH, and these clusters were used as operational taxonomic units^33^ (OTUs) in downstream analyses, approximating species-level groupings and collapsing individual, potentially intragenomic sequence variants^34,35^. We thus retain rare variants at the denoising step and summarise diversity at the 98%-similarity OTU level, rather than treating each denoised exact sequence variant as a separate analytical unit, because long amplicons contain many singleton and other low-abundance variants that may represent genuine rare taxa, and removing them at the denoising step could lead to an underestimation of rare diversity^34,36^. The 98% similarity threshold represents a pragmatic compromise across divergent eukaryotic lineages and may affect richness estimates in groups with different rates of ITS evolution, but implementing lineage-specific clustering thresholds at this scale is currently not feasible.

Representative ITS sequences of each OTU, as well as the corresponding small (SSU) and large (LSU) subunit fragments of the same representative, were queried with BLASTn v.2.16.0+^37^ against the EUKARYOME database, retaining the ten best hits. Extensive manual curation was performed using taxon-specific E-value and sequence similarity thresholds^5^. Additionally, long-read chimeras were detected by comparing region-specific taxonomic assignments across the SSU, ITS, and LSU segments of each read. Reads were classified as chimeric when these segments yielded incongruent higher-level placements, as determined by manual inspection of the top BLAST hits, using phylum-level disagreement for Ascomycota and Basidiomycota and order-level disagreement for other taxa. Disagreement restricted to lower taxonomic ranks was not treated as sufficient evidence of chimerism. Non-target OTUs (e.g., sequences of archaeal or bacterial origin) were removed. Samples with <500 total reads or identified as potentially contaminated (i.e., had >30% of reads corresponding to molds or shared OTUs with negative or positive controls) were excluded from the final dataset. To reduce the amount of unidentified OTUs and improve the precision of chimera detection, we also amplified and sequenced an ultra-long rRNA gene fragment spanning the SSU V3 through ITS through LSU D8 from >900 soil samples using the primers EUK575F (5′-TASCYGYGGTAAYWCCAGC-3′) and 21R (5′-AGAGACGAGGCATTTGGCTAC-3′)^38,39^ on PacBio Sequel II and Revio platforms.

To complement EUKARYOME-based taxonomic annotations of fungal OTUs, we additionally performed UNITE Species Hypotheses (SH) matching^40–42^ to assign persistent DOI-registered identifiers to representative ITS sequences. The analysis was performed using the SH-matching analysis tool v.2.0.3, which places query ITS sequences into existing SHs in the UNITE database v.10.0^43^ or assigns to new SHs with preliminary codes. The stable identifiers enable unambiguous cross-study referencing of “dark taxa”^44^, remain valid as taxonomy changes, and facilitate reporting and reuse of DNA-derived occurrences. Because SHs are integrated into the GBIF (https://www.gbif.org/) taxonomic backbone, SH-based occurrences from this study can be directly compared, aggregated, and cross-linked with GBIF records and other datasets.

## Data Records

The GloSED dataset is available at Zenodo^45^ (versioned release archive DOI: 10.5281/zenodo.17827890). The Zenodo deposit is organised as a set of files, including: (1) detailed sample metadata with environmental variables (‘GloSED__Sample_metadata.xlsx’), (2) quality-filtered OTU sequences in FASTA format (‘GloSED__OTU_sequences.fasta.gz’), (3) sample-by-OTU abundance matrices (‘GloSED__OTU_table.tsv.zip’ and ‘GloSED__OTU_table.parquet’), and (4) curated taxonomic annotations (‘GloSED__Taxonomy.tsv.zip’ and ‘GloSED__Taxonomy.parquet’).

‘GloSED__Sample_metadata.xlsx’ is the main metadata table and contains one row per sample. The accompanying legend sheet defines all column names and measurement units. The key linking fields are ‘SampleID’ and ‘TUE code’, where the latter is the accession of the physical soil sample in the University of Tartu collection. The metadata describe when and where each sample was collected, including geographic coordinates, elevation, locality, administrative unit, land-cover type, and co-occurring plant taxa. The table also includes measured soil properties, including pH, total soil carbon and nitrogen, C:N ratio, stable isotope abundances, as well as nutrient concentrations for phosphorus, potassium, calcium, and magnesium.

OTU abundance data are provided as ‘GloSED__OTU_table.tsv.zip’ and ‘GloSED__OTU_table.parquet’. The Parquet file stores the abundance information in long format with the fields ‘OTU’, ‘SampleID’, and ‘Abundance’, whereas the tab-delimited file contains sample-by-OTU abundance matrix in wide format. Taxonomic annotation is provided in ‘GloSED__Taxonomy.tsv.zip’ and ‘GloSED__Taxonomy.parquet’. These files contain the OTU identifier, the representative sequence, accession number of the best EUKARYOME match (column ‘AccID’) and its alignment statistics (sequence identity percentage, coverage, E-value, and BLAST bitscore), curated taxonomic ranks from ‘Kingdom’ to ‘Species’, and the UNITE fields ‘SH_30’ to ‘SH_05’, which record persistent fungal species-hypothesis identifiers at progressively finer similarity thresholds (from 3% to 0.5%). Files in Apache Parquet^46^ format provide a programming-language-independent columnar file structure that allows efficient storage and high-performance analytical operations, whereas TSV files offer broader compatibility. Representative OTU sequences are supplied separately in ‘GloSED__OTU_sequences.fasta.gz’, with FASTA headers matching the OTU identifiers (40-character hexadecimal strings based on the SHA1 hash of the primer-trimmed sequence prior to ITSx extraction).

For common downstream workflows, the same core data are additionally distributed as a biological observation matrix (BIOM) v.2.1 format file^47^ for QIIME2^48^ integration (file ‘GloSED__BIOM.biom’) and as a *phyloseq*^49^ object for R-based analyses (‘GloSED__phyloseq.RData’). ‘Contributors.xlsx’ provides the contributor list associated with the release. Raw demultiplexed FASTQ files are available from the European Nucleotide Archive (ENA) under project PRJEB103811^50^.

## Data Overview

**Fig. 1:**
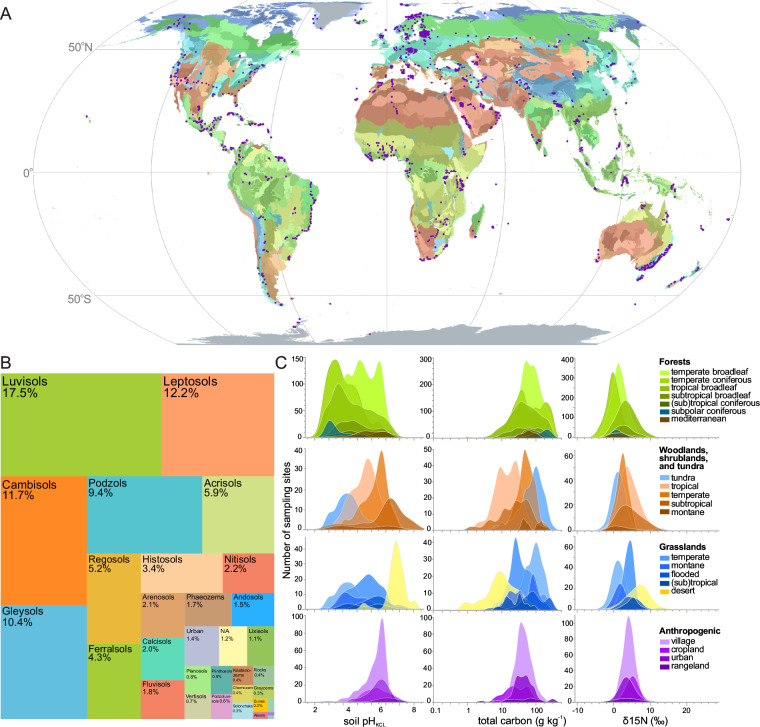
Examples of environmental gradients represented by GloSED (**A**) GloSED sampling sites are distributed across 351 terrestrial ecoregions. Dots mark sampling sites; ecoregions within the same biome share color hues. (**B**) GloSED represents soil types in proportion to their global distribution. (**C**) Soil pH, total carbon, and δ15N, measured directly in GloSED samples, have broad distributions within and across land-cover types.

The GloSED is a structured dataset including 4,147 samples from 4,063 sampling sites worldwide (Fig. 1A), analysed using standardised field and laboratory protocols. Each site record contains geographic coordinates, sampling date, land-cover type assigned by the field collector, and plot-level information on the dominant plants, mostly for woody land cover types. For nearly 95% of sites, directly measured soil properties are recorded, including pH, total carbon and total nitrogen contents (g kg^−1^), δ^15^N (‰), δ^13^C (‰), as well as available phosphorus and potassium, and exchangeable magnesium and calcium contents (each mg kg^−1^).

All samples are associated with read counts for each of 988,824 operational taxonomic units (OTUs) of eukaryotic organisms. Each OTU is taxonomically annotated using the EUKARYOME reference database, assigned a species hypothesis (SH) identifier using the UNITE reference database, and linked to quality-curated representative sequences of full-length ITS and 18S-V9 regions. Median observed OTU richness and effective number of OTUs per sample were 829 (Q1-Q3: 526–1213) and 184 (Q1-Q3: 106–280), respectively.

## Technical Validation

### Methods for technical data validation

All analyses, and visualization were conducted in R v.4.5.2^51^. For high-performance operations on large datasets, we performed data manipulation and processing using the data.table v.1.17.8^52^ and Apache arrow v.21.0.0^53^ packages. Taxonomic resolution and sequencing completeness metric were assessed with the metagMisc package v.0.5.0^54^. Observed abundance-based sample coverage was used to estimate sequencing completeness after correcting singleton counts with a modified Good-Turing estimator^55^. The effective number of OTUs was calculated as the exponential of the Shannon diversity index, following Jost^56^.

Spatial data were processed using the sf package v.1.0-21^57^. For map visualizations, we used the Equal Earth projection^58^. Sample administrative location was assigned using GADM v.4.1 boundary polygons^59^, biomes and ecozones were determined following Loidi *et al*.^60^, and ecoregions and soil types were assigned using the Ecoregions 2017 dataset^61^ and Harmonized world soil database v.2.0^62^, respectively. We quantified environmental novelty of unsampled locations using a dissimilarity index (DI) following the area-of-applicability framework^63^. The DI was calculated from 14 Z-standardized, importance-weighed bioclimatic and edaphic predictors identified as key drivers of soil fungal communities^4^.

### Sampling consistency and representativeness

A consistent sampling design is a fundamental prerequisite for obtaining unbiased biodiversity data^64^. For soil biota in particular, differences in sampling time, effort, area, depth at which soil is collected, and compositing strategy can cause orders-of-magnitude variation in biodiversity estimates^65,66^. Similar effects arise from inconsistencies in analytical protocols^65–67^. GloSED minimizes these potential biases by applying identical sampling and analytical procedures across all sites.

GloSED applies a 5 cm sampling depth to target the most biologically active soil layer where root density, microbial activity, and organic matter content are typically the highest^68^. The relatively large sampling area (2,500 m²) – one of the largest commonly used in soil microbial ecology – ensures comprehensive representation of local biota, including soil macrofauna such as annelids and arthropods.

Furthermore, on-site descriptions of land cover by collectors coupled with direct measurements of soil chemical properties, provide data that are more representative of real-world conditions than those derived from modelled or predicted datasets.

### Bioinformatic validation

For the database, more than 73 million HiFi PacBio DNA reads were generated from 122 sequencing runs targeting full-length ITS and 18S-V9 variable regions. During bioinformatic processing, to ensure high data quality, we paid special attention to homopolymer errors, PCR-mediated chimeras, and index switching - technical artefacts that are common in amplicon sequencing but typically undetectable by default pipelines^18,69^. Comparison with reference sequences revealed more than one million chimeric reads, and *de novo* analysis combined with manual curation flagged additional 58,119 chimeras. Index switching rate was within acceptable thresholds (0.02%). All the chimeric reads and false assignments were removed prior to downstream analyses. The 4,147 samples that passed all stages of technical control yielded more than 27 million high-quality sequences with a median number of 5,193 sequences per sample (IQR: 2,766–8,409). Median sample coverage was 0.95 (Q1–Q3: 0.92–0.97), suggesting a high sequencing completeness for most samples.

### Geographical and environmental coverage

**Fig. 2:**
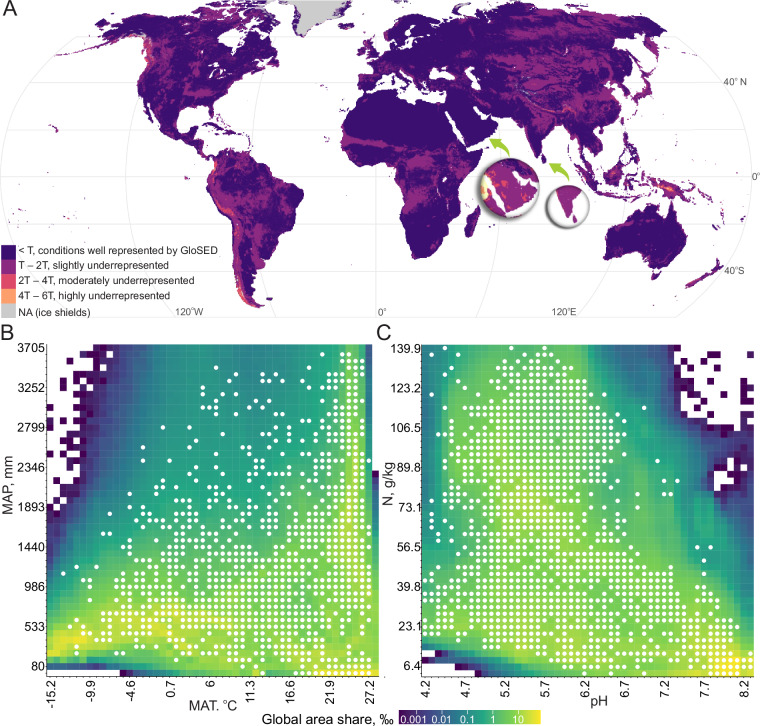
Examples of GloSED coverage of global environmental space. (**A**) Based on 14 climatic, vegetation, and edaphic predictors of total soil fungal diversity^4^, 71.6% of global terrestrial area falls within the GloSED sampling range (dark purple), and 26.3% deviates from this range by less than twofold (purple). Red and orange colors (0.72% and 0.04% of total area) denote the most environmentally novel terrestrial areas relative to the currently sampled space. (**B,C**) show that GloSED captures the majority of the global range of mean annual temperature (MAT) with precipitation (MAP), and topsoil pH with total nitrogen concentration, respectively. Dots mark environmental bins represented by GloSED samples.

Sufficient geographical and environmental coverage is a prerequisite for a global dataset suitable for cross-regional comparisons and predictive modelling^63,70^. The GloSED spans from –73.0° to 79.6° latitude, encompassing all terrestrial climate zones (as per Beck *et al*.^71^), 351 terrestrial ecoregions and all major soil types with broad distributions of soil properties (Fig. 1). Previous global models of soil biodiversity identified regions with high uncertainty in predictions, as these regions differ substantially in environmental conditions from the data observed at sampling locations^4,72^. In a recent sampling campaign, we specifically targeted such regions. For instance, substantial contributions of samples from Australia, India, Saudi Arabia, Algeria, Liberia, Kazakhstan, Suriname, and Uruguay improved the representativeness of hot and temperate deserts as well as tropical and grassland biomes (Fig. 2) – large areas that have shown the greatest variability in soil fungal biodiversity predictions across studies.

### Taxonomic coverage and resolution

**Fig. 3:**
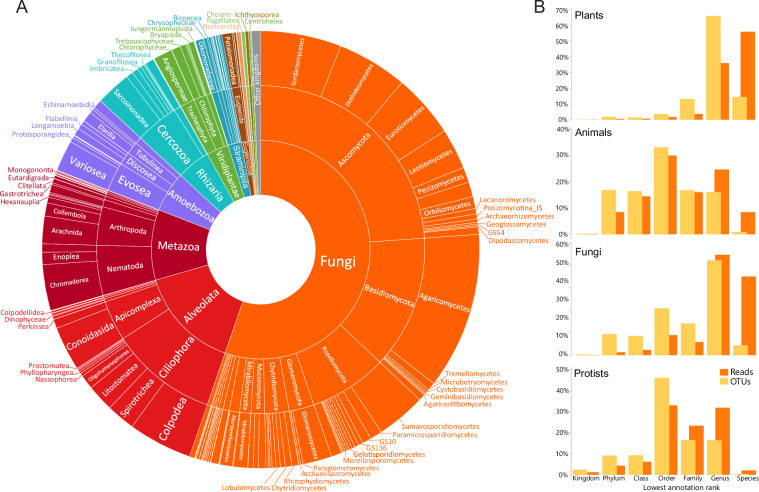
GloSED taxonomic coverage and resolution. (**A**) GloSED taxonomic coverage and (**B**) resolution allows for functional annotation of the majority of soil eukaryotes. Sector angular span denotes the percentage of operational taxonomic units (OTUs) belonging to taxonomic groups. Due to limited space, not all taxa are labelled.

Owing to the use of universal eukaryotic markers (full-length ITS and 18S-V9 regions), GloSED is the only pan-domain soil dataset, with a standardized design, currently available. It encompasses 546,665 fungal OTUs (73.6% of reads), 316,021 protistan OTUs (16.8% of reads), 103,021 animal OTUs (3.4% of reads), and 19,186 plant OTUs (5.7% of reads) (Fig. 3A).

Over 40% of fungal OTUs were taxonomically annotated to the genus level (Fig. 3B). As is typical for soil fungal communities, Ascomycota (235,918 OTUs) and Basidiomycota (130,119 OTUs, with 88% belonging to Agaricomycetes) represent the richest phyla. Notably, GloSED taxonomic annotation based on EUKARYOME, curated by leading specialists in fungal and protist taxonomy^73–76^, achieves exceptional resolution for underexplored lineages that may account for a substantial part of the community. For instance, non-Dikarya fungi comprise 180,367 OTUs (33% of total fungal diversity), including 76,113 OTUs from Rozellomycota (also known as Cryptomycota), 24,388 OTUs from Glomeromycota, 23,545 OTUs from Chytridiomycota, 13,319 OTUs from Mucoromycota, and 10,088 OTUs from Mortierellomycota. Owing to a substantially redesigned bioinformatic workflow, the sequence representatives in GloSED differ in both composition and number from those in GSMc.

Among animals, almost half of the data belong to Nematoda (45.6% of metazoan reads; 48,674 OTUs), followed by Arthropoda (36.0% of reads; 37,545 OTUs) and Annelida (10.3% of reads; 3,432 OTUs). Other represented groups include Gastrotricha (2.3% of metazoan reads; 7,097 OTUs), Tardigrada (2.9%; 2,522 OTUs), Rotifera (0.6%; 1,624 OTUs), and Platyhelminthes (1.6%; 1,511 OTUs). Over 16% of animal OTUs are taxonomically annotated to the family level and over 32%, to the order level.

Protistan data span more than 30 phyla, with 74.9% of reads belonging to Alveolata (150,508 OTUs), including almost 40,000 Apicomplexa OTUs - a largely parasitic group associated with major public-health burdens and substantial economic losses^77^. Almost 7% of protist reads belong to each of Amoebozoa (59,179 OTUs) and Rhizaria (49,195 OTUs), 4.7% to green algae (13,120 OTUs), and 3.8% to Straminipila (19,580 OTUs) with 3,308 OTUs belonging to Oomycota – the most economically damaging protist group^78^. Almost 50% of protist OTUs are identified to the order level.

Tracheophyta (vascular plants) cover 73.1% of plant reads (14,030 OTUs, with 98% belonging to angiosperms), and Setaphyta (bryophytes) 26.9% (5,156 OTUs). Over 65% of plant OTUs are annotated to the genus level. This enables the inclusion of plants - key ecosystem engineers – alongside soil microbes, reflecting their reciprocal influence as both drivers and responders within soil biodiversity patterns.

The broad taxonomic coverage of GloSED enables integrative research of soil eukaryotes including primary producers, decomposers, consumers, and parasites. The achieved taxonomic resolution allows functional annotation for a substantial proportion of the GloSED OTUs using databases such as FungalTraits^79^ for fungi, Nemaplex^80^ for nematodes, FunctionalTraitsAmoebozoa^81^ for Amoebozoa and Rhizaria, and TRY for plants^82^.

### Cross-dataset comparability

As taxonomy constantly advances, OTUs must be traced across multiple data sources supporting their re-annotation. This is achieved with the UNITE species hypothesis (SH) system – a standardised digital framework for discovering and communicating fungal species, particularly those identified from environmental DNA^42^. In the GloSED dataset, nearly half of all fungal OTUs are assigned to existing SHs at the 97% similarity cut-off, with approximately 20% assigned at the 99.95% threshold. Each SH is associated with a stable DOI-linked reference integrated with the PlutoF and GBIF taxonomic backbones.

## Usage Notes

Raw sequencing data can be re-analysed using the NextITS workflow. The source code of this bioinformatics pipeline and its documentation are available under MIT license at https://github.com/vmikk/NextITS and https://Next-ITS.github.io/, respectively. For reproducible execution, containerised environments are hosted at Docker Hub (https://hub.docker.com/r/vmikk/nextits) and Singularity library (https://cloud.sylabs.io/library/vmiks/nextits/nextits). Processing the full GloSED dataset on the University of Tartu high-performance computing (HPC) cluster using AMD EPYC 7702 processors required approximately 13,300 CPU hours, with wall-clock runtime depending on the execution profile and degree of parallelisation. ITSx represented the main bottleneck in the pipeline, and the BLAST searches required an additional approximately 20,600 CPU hours. To support data reuse, we followed the guidelines of Hug *et al*.^83^.

## Data Availability

The dataset and sample metadata are available on Zenodo^45^ (https://zenodo.org/records/17827890), and the raw sequencing data have been deposited in the European Nucleotide Archive (ENA) under project accession PRJEB103811 (sample accession numbers ERS27941879 - ERS27946063; sequence accession numbers ERR15957609 - ERR15964175)^50^.

## Code availability

Analysis scripts for manuscript figures and supporting analyses are deposited at GitHub (https://github.com/Mycology-Microbiology-Center/GloSED).
